# Induction of ErbB-3 Expression by α6β4 Integrin Contributes to Tamoxifen Resistance in ERβ1-Negative Breast Carcinomas

**DOI:** 10.1371/journal.pone.0001592

**Published:** 2008-02-13

**Authors:** Valentina Folgiero, Paolo Avetrani, Giulia Bon, Selene E. Di Carlo, Alessandra Fabi, Cecilia Nisticò, Patrizia Vici, Elisa Melucci, Simonetta Buglioni, Letizia Perracchio, Isabella Sperduti, Laura Rosanò, Ada Sacchi, Marcella Mottolese, Rita Falcioni

**Affiliations:** 1 Department of Experimental Oncology, Regina Elena Cancer Institute, Rome, Italy; 2 Department of Medical Oncology, Regina Elena Cancer Institute, Rome, Italy; 3 Department of Pathology, Regina Elena Cancer Institute, Rome, Italy; 4 Scientific Direction, Regina Elena Cancer Institute, Rome, Italy; University of Hong Kong, China

## Abstract

**Background:**

Tamoxifen is still the most widely used drug in hormone therapy for the treatment of breast cancer. Its benefits in adjuvant treatment are well documented in controlled and randomized clinical studies, which have demonstrated an increase in disease-free intervals of patients with positive hormonal receptors. However, the mechanisms involved in endocrine resistance are not clear. Laboratory and clinical data now indicate that bi-directional molecular cross-talk between nuclear or membrane ER and growth factor receptor pathways may be involved in endocrine resistance. We recently found a functional interaction between α6β4 integrin and ErbB-3 receptor to maintain the PI3K/Akt survival pathway of mammary tumour cells. We sought to improve understanding of this process in order to provide the involvement of both receptors insight into mechanism of Tamoxifen resistance.

**Methods and Findings:**

Using human breast cancer cell lines displaying different levels of α6β4 and ErbB-3 receptors and a series of 232 breast cancer biopsies from patients submitted to adjuvant Tamoxifen monotherapy for five years, we evaluated the functional interaction between both receptors in relationship to Tamoxifen responsiveness. In mammary carcinoma cells, we evidenced that the α6β4 integrin strongly influence Akt phosphorylation through ErbB-3 protein regulation. Moreover, the ErbB-3 inactivation inhibits Akt phosphorylation, induces apoptosis and inhibits *in vitro* invasion favouring Tamoxifen responsiveness. The analysis of human tumors revealed a significant relationship between α6β4 and ErbB-3 in P-Akt-positive and ERβ1-negative breast cancers derived from patients with lower disease free survival.

**Conclusions:**

We provided evidence that a strong relationship occurs between α6β4 and ErbB-3 positivity in ERβ1-negative breast cancers. We also found that the association between ErbB-3 and P-Akt positivity mainly occurs in ERβ1-negative breast cancer derived from patients with lower DFS indicating that both receptors are clinically relevant in predicting the response to Tamoxifen.

## Introduction

In many breast cancer (BC), activation of the phosphatidylinositol 3-kinase (PI3K) pathway may deeply reduce the efficacy to targeted therapies [1]–[3]. In the last few years, a strong activation of the PI3-K/Akt signaling pathway was observed in tumor cells that express high levels of integrin α6β4, a laminin receptor implicated in tumor progression and invasion [4]–[9]. The involvement of this integrin in tumor progression is supported by large experimental evidence. In mammary and ovary carcinoma cell lines, α6β4 integrin associates with ErbB-2 overexpression and co-operates to promote a PI3K-dependent invasion and survival [10], [6]. In MMTV-Neu mice, the introduction of a targeted deletion of the β4 cytoplasmic domain revealed that β4 integrin promotes tumor progression cooperating with ErbB-2 signaling [11]. Inactivation of α6β4 integrin by RNA interference inhibits tumor growth both *in vitro* and *in vivo* [12]–[14] and strongly reduces the activity of the PI3K pathway inducing apoptosis upon hormone deprivation and TAM treatment in MCF7 BC cells [12]. In addition, we have recently evidenced that the α6β4-induced PI3K-dependent survival pathway of two different BC cell lines is due to the capability of α6β4 integrin to enhance ErbB-3 expression. This enhancement results in an increase of ErbB-2/ErbB-3 heterodimerization and consequently in the activation of the PI3K survival pathway [15]. Collectively, these studies suggest a strong cooperation between α6β4 integrin and EGFR family members in mammary tumors and highlight a pathway by which this integrin might contribute to BC tumorigenicity and responsiveness to treatments.

BC remains one of the most heterogeneous tumors in terms of capability to give metastases, expression of hormone receptors and responsiveness to therapies and is the first cause of death for women aged 35–45 years [16]. Tamoxifen (TAM) is still the most widely used drug in hormone therapy for the treatment of this neoplasia. Its benefits in adjuvant treatment and metastatic disease are well documented in controlled and randomized clinical studies, which have demonstrated an increase in disease-free intervals and overall survival of patients with positive hormonal receptors [17]. However, endocrine therapies do not always work in patients, despite the presence of hormone receptors in their tumors [18]. Originally, only estrogen receptor (ER) α and progesterone receptor (PgR) were thought to be involved in hormone signaling. However, a second ER, termed ERβ, was subsequently discovered, adding another dimension of complexity to the regulation of hormone response [19]–[20]. Insights into the mechanisms of endocrine therapy resistance, although still cause for debate, have come from several studies concerning the biology of ERs and the various signaling pathways in the cell with which they communicate. Laboratory and clinical data now indicate that bidirectional molecular cross-talk between nuclear or membrane ER and growth factor receptor pathways may be involved in endocrine resistance [21]. An understanding of these ER activities at the molecular level may yield new strategies to prevent or overcome resistance to TAM and other forms of treatment.

In the present work, using ER-positive human BC cell lines, we investigated the functional interaction between α6β4 and ErbB-3 proteins in relationship to TAM responsiveness. In addition, with the aim to translate our *in vitro* study to an *in vivo* model, we carried out immunohistochemical (IHC) analysis to evaluate the functional relationship between desease-free survival (DFS) and expression of α6β4, ErbB-2, ErbB-3, P-Akt and ERβ1 in a retrospective series of 232 ERα and/or PgR positive BCs derived from patients which had been homogeneously submitted to adjuvant TAM monotherapy. Combining our analyses, we provide evidence that α6β4 expression is functionally associated with ErbB-3 and P-Akt molecules *in vitro*. However, even though α6β4 expression *in vivo* is still strongly associated with ErbB-3 positivity and ERβ1 negativity, it does not influence patient outcome. Interestingly, we report for the first time a strong association of ErbB-3 and P-Akt positivity that mainly occurs in ERβ1 negative BC derived from patients with lower DFS. This result suggests that both receptors are clinically relevant in predicting the response to Tamoxifen treatment.

## Results

### Expression of β4, ErbB-2, ErbB-3, ERα and ERβ receptors in mammary tumor cell lines

**Figure 1:**
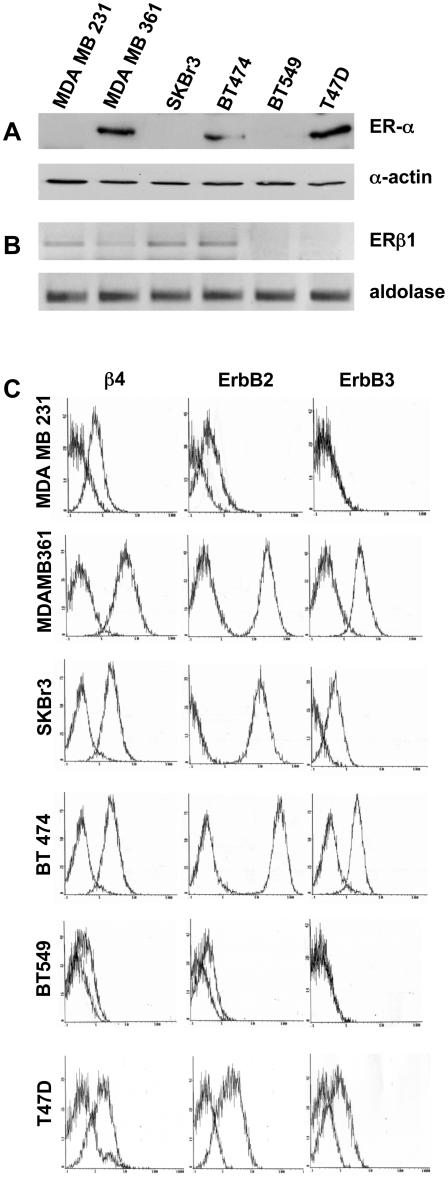
Expression of β4, ErbB-2, ErbB-3 and ERα and β1 receptors in mammary tumor cell lines. A. The expression of ERα was evaluated by western blot analysis. The anti-actin Ab was used to validate equivalent loading protein. B. ERβ1 expression was evaluated by RT-PCR from total mRNA extracted from the indicated cell lines using primers specific for human ERβ1 and the housekeeping aldolase genes. C. Mammary tumor cell lines MDA-MB 231, MDA-MB 361, SKBr3, BT474, BT549 and T47D were analyzed by FACS to reveal the expression level of β4 integrin subunit, ErbB-2 and ErbB-3 receptors.

We first evaluated the expression level of ERα and ERβ, β4 integrin subunit, ErbB2, and ErbB-3 in a series of human mammary tumor cell lines including MDA-MB 231, MDA-MB 361, SKBr3, BT474, BT549, and T47D. Analysis of ERα by Western blotting (Figure 1A) and ERβ1 by RT-PCR, using specific primers to detect ERβ1 mRNA, (Figure 1B) showed that BT549 cells were negative for both ERs whereas the other cell lines were positive for at least one ER. Then, the expression of ERβ1 protein was evaluated by immunocytochemistry (Figure S1). The data obtained confirmed the expression of ERβ1 protein in each cell line that resulted positive for ERβ1 mRNA. As expected, the analysis of the other receptors by cytofluorimetry showed that MDA-MB 361, SKBr3 and BT474 and T47D cells express considerable levels of ErbB-2 protein (Figure 1C) [22]. Moreover, the same cells express β4 and ErbB-3 proteins at comparable levels, whereas BT549 and MDA-MB 231 cells displaying low levels of ErbB-2 and β4 proteins were also ErbB-3 negative, supporting our recent finding that β4 overexpression regulates ErbB-3 protein at translational level [15].

#### The regulation of ErbB-3 expression by α6β4 influences AKT activation

**Figure 2:**
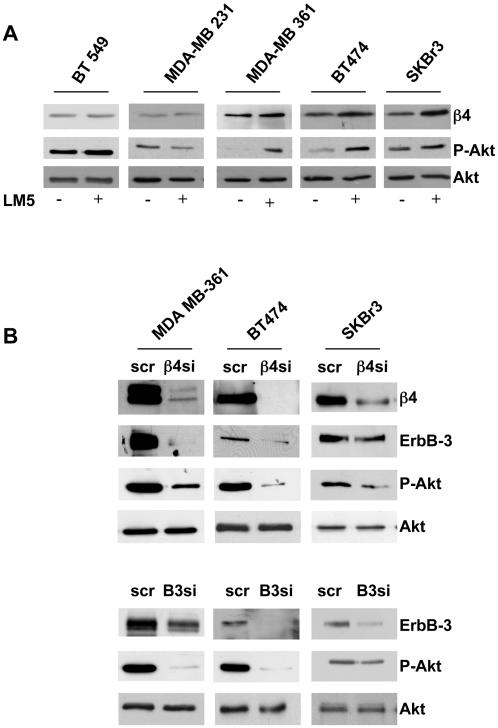
The α6β4 influence Akt activation by ErbB-3. A. BT549, MDA-MB 231, MDA-MB 361, BT474 and SKBr3 cells were serum-starved for 24 hrs and then the cells were spread onto LM5 and extracted in detergent. Equivalent amounts of protein were separated by SDS-PAGE and analyzed by immunobloting to evaluate the relative expression of β4 and phospho-Akt. Total-Akt Ab was used to validate equivalent loading of protein in each lane. B. MDA-MB 361, BT474 and SKBr3 cells were transiently transfected for 48 hrs with either scrambled or specific β4-shRNA and ErbB-3 siRNA. The cells were then serum-starved for 24 hrs and extracted in detergent. Equivalent amounts of protein were separated by SDS-PAGE and analyzed by immunobloting to evaluate the relative expression of β4, ErbB-3 and phospho-Akt. Hsp70 Ab was used to validate equivalent loading of protein in each lane.

Given that α6β4 integrin is the receptor for laminin 5 (LM5) and, as we previously demonstrated, ligation of the integrin to this substrate enhances PI3K signaling, we first verified the level of Akt phosphorylation upon stimulation in the mammary tumor cell lines. To this end, MDA-MB 361, BT474, SKBr3, BT549 and MDA-MB 231 cells were spread onto LM5 for 20 minutes and the level of Akt activity was evaluated by Ser473 phosphorylation. As reported in Figure 2A, a strong enhancement of Akt phosphorylation was detectable in the cells expressing α6β4, ErbB-2 and ErbB-3 receptors (i.e., MDA-MB 361, BT474 and SKBr3 cells) while, it did not occur in cells expressing low levels of β4, ErbB-2 and undectable level of ErbB-3 (i.e., BT549 and MDA-MB 231) (Figure 2A). As expected, after 60 minutes of LM5 stimulation, the phosphorylation of Akt returned to the basal levels (data not shown).

To confirm the essential role of ErbB-3 protein in the activation of Akt by α6β4, a β4 shRNA (β4si) or an ErbB-3 siRNA (B3si) were expressed in MDA-MB 361, BT474 and SKBr3 cells, as previously described [15]. As expected, depletion of β4 resulted in a strong reduction of β4 compared to the levels found in scramble (scr) control cells. Of interest, β4 depletion also caused a strong reduction of ErbB-3 expression and Akt phosphorylation (Figure 2B, upper panel). Moreover, ErbB-3 depletion resulted in a strong reduction of ErbB-3 expression and, at the same time, of Akt phosphorylation (Figure 2B, lower panel). Since α6β4 regulates ErbB-3 level and the depletion of either β4 or ErbB-3 proteins resulted in a strong inhibition of Akt activation, the data confirm the essential role of ErbB-3 in the activation of Akt by α6β4 integrin in mammary tumor cells (Figure 2B).

#### ErbB-3 depletion causes apoptosis and inhibits *in vitro* invasion favoring TAM responsiveness

**Figure 3:**
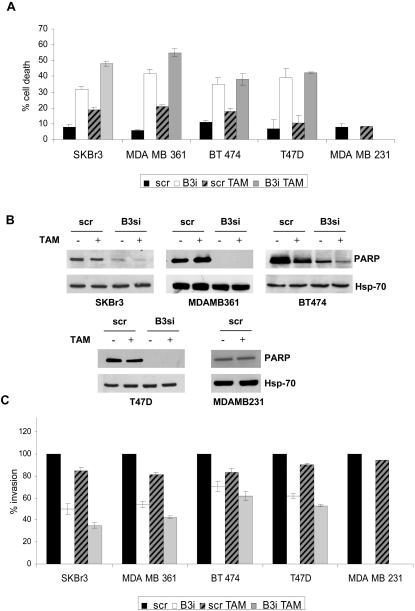
ErbB-3 expression influences survival and invasion of mammary tumor cells treated under TAM treatment. A. SKBr3, MDA-MB 361, BT474, T47D and MDAMB231 cells after three days of hormone deprivation were transiently transfected with either scrambled or specific ErbB-3 siRNA. Where specified, 24 hrs after transfection scrambled and ErbB3 interfered cells were pre-incubated for 24 hours at 37°C with TAM 2.5 µM. 48 hours following transfection, the cell death was evaluated by Trypan-blue exclusion. Statistical differences were evaluated by T test (p<0.05). B. Equivalent amounts of total cell lysate derived from the cell lines described in A were separated by SDS-PAGE and analyzed by immunobloting to evaluate the expression level of PARP cleavage. Hsp70 Ab was used to validate equivalent loading of protein in each lane. C. SKBr3, MDA-MB 361, BT474, T47D and MDAMB231 cells transfected as described in A were assayed for their ability to invade matrigel in the absence of hormone and under TAM treatment. Statistical differences were evaluated by T test (p<0.05).

To further evaluate the function of ErbB-3 in the PI3K survival pathway, we analyzed cell death and apoptosis in the absence of hormones and under TAM treatment of ErbB3 positive (SKBr3, MDA-MB 361, BT474 and T47D) and ErbB3 negative (MDAMB231) cell lines. As shown in Figure 3A and 3B, in the absence of hormones, depletion of ErbB-3 protein caused *per se* a strong cell death compared to scr cells (SKBr3/B3i 32% vs SKBr3/scr 8%, p = 0.001; MDA MB361/B3i 42% vs MDA MB361/scr 6%, p<0.0001; BT474/B3i 35% vs BT474/scr 11%, p = 0.04; T47D/B3i 39% vs T47D/scr 7%, p = 0.04). Cell death was further increased by TAM treatment (SKBr3/B3si/TAM 48% vs SKBr3/scr/TAM 19%, p<0.0001; MDA MB361/B3si/TAM 55% vs MDA MB361/scr/TAM 21%, p<0.0001; BT474/B3si/TAM 38% vs BT474/scr/TAM 18%, p = 0.02; T47D/B3si/TAM 42% vs T47D/scr/TAM 10%, p = 0.005) as also assessed by cleavage of PARP, a marker of apoptotic death (Fig. 3B). The results we obtained on cell death and apoptosis on T47D cells strongly reinforce our hypothesis that ErbB-3 sustains the survival function of mammary tumor cells in the absence of hormone stimuli. Indeed, this cell line is negative for ERβ1 expression, does not respond to TAM treatment, and undergoes apoptosis only upon ErbB-3 depletion. MDAMB231 cells that are ErbB-3 negative, even if are ERβ1 positive, do not respond to TAM treatment and proliferate and survive as well as untreated cells (p = 0.82) (Fig. 3A,B), indicating that these cells have developed other survival pathway(s).

It is widely reported that TAM resistance and, as a consequence, tumor progression may be also due to PI3K activation [23]. In order to understand the role of ErbB-3 in the invasion process, we evaluated the invasive capability of scr and ErbB-3-depleted SKBr3, MDA-MB-361, BT474, T47D cells in the absence of hormones and upon TAM treatment. As shown in Figure 3C, depletion of ErbB-3 protein caused *per se* a strong inhibition of the invasion compared to scr cells (percent of invasion: SKBr3/B3si 50% vs SKBr3/scr 100%, P = 0.001; MDA MB361/B3si 54% vs MDA MB361/scr 100%, p<0.0001; BT474/B3si 70% vs BT474/scr 100%, p = 0.03; T47D/B3si 62% vs T47D/scr 100%, p = 0.04). The inhibition of the invasion in ErbB-3-depleted cells further increased following TAM treatment compared to scr cells (percent of invasion: SKBr3/B3si/TAM 35% vs SKBr3/scr/TAM 85%, p<0.0001; MDA MB361/B3si/TAM 42% vs MDA MB361/scr/TAM 82%, p<0.0001; BT474/B3si/TAM 61% vs BT474/scrTAM 83%, p = 0.04; T47D/B3si/TAM 50% vs T47D/scr/TAM 90%, p<0.001). As expected, TAM treatment does not alters the capability of MDAMB231 cells to invade matrigel (p = 0.06) (Figure 3C). Representative invading stained cells are showed on Figure S2. Collectively, these data indicate a role of ErbB-3 protein in the mechanisms that regulate the invasion of mammary tumor cells. Since we have previously demonstrated that β4 depletion reduces the responsiveness of mammary tumor cells to TAM treatment, our data also suggest that a cooperative signaling between ErbB-3 and α6β4 integrin could influence resistance to hormone therapy *in vivo*.

### Immunohistochemical analysis of β4 integrin subunit, ErbB-3, ErbB-2, P-Akt, and ERβ1 in human primary BC

**Figure 4:**
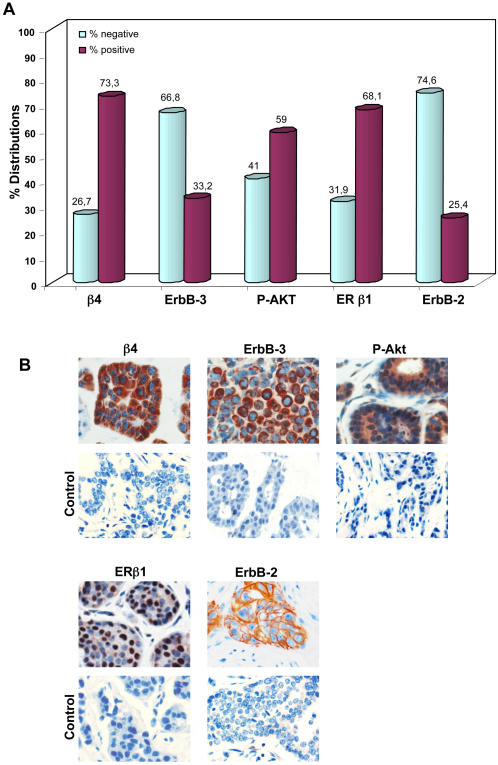
Immunohistochemical analysis of β4, ErbB-3, P-Akt, ERβ1 and ErbB-2 in 232 primary BC. A. Distribution (%) of the bio-pathological factors β4 integrin subunit, ErbB-3, P-Akt(ser473), ERβ1 and ErbB-2 in 232 TAM treated breast cancers. B. Representative immunohistochemically positive cases for β4, ErbB-3, P-Akt(ser473), ERβ1 and ErbB-2 protein detection and control tissue sections.

To verify whether the functional interaction between α6β4 integrin and ErbB-3 receptor also occurred *in vivo*, we studied 232 biopsies of BC patients surgically treated at our Institute and submitted to adjuvant TAM therapy. The detailed clinicopathological characteristics of the patients are described in Table 1. These tumors were first analyzed by IHC for the expression of β4 integrin subunit, ErbB-3, ErbB-2, ERβ1 and P-Akt expression. As summarized in Figure 4A, of the 232 cases analyzed, β4 exhibited a strong homogeneous (score 2) or heterogeneous (score 1) immunoreaction in 170 BC (73,3%). 77 BC (33,2%) overexpressed ErbB-3 and 158 (68,1%) were ERβ1 positive. Moreover, we found that 136 BC (59%) were P-Akt positive, while 59 (25,4%) were positive for ErbB-2. Representative immunohistochemically positive cases for β4, ErbB-3, P-Akt, ERβ1 and ErbB-2 and control tissue sections are shown in Figure 4B.

**Table 1:** Clinicopathological characteristics of 232 invasive breast carcinomas TAM treated

CHARACTERISTIC		%
**Number of patients**	**232**	
**Mean age**	**63**	
**Menopausal**		
Pre	25	10.8
Post	207	89.2
**Histotype**		
Invasive ductal carcinoma	193	83.2
Invasive lobular carcinoma	28	12.1
Tubular carcinoma	7	3
Papillary carcinoma	4	1.7
**Tumor size**		
T1	176	79.9
T2	51	22
T3,T4	5	2.1
**Lymph node status**		
Negative	193	83.2
Positive	39	16.8
**Grading**		
G1	53	22.8
G2	141	60.8
G3	38	16.4
**ERα**		
Negative (≤10%)	25	10.8
Positive (>10%)	207	89.2
**PgR**		
Negative (≤10%)	55	23.7
Positive (>10%)	177	76.3

* range 39–95

### Relationship among β4 integrin subunit, pathological and biological parameters

Table 2 summarizes the associations between β4 expression and biopathological factors in our series of 232 BC patients. We found that all tumors, which were positive for ErbB-3 receptor, showed a higher score in β4 expression, β4 immunoreaction being significantly associated to ErbB-3 (p = 0.003). Interestingly, we also found that the majority of high β4-positive tumors were ERβ1-negative (p<0.0001). In contrast, β4 was not significantly related to P-Akt, ErbB-2 protein and any conventional pathological parameters, namely tumor size, grading and nodal status.

**Table 2:** Relationship between integrin β_4 _expression and biopathological factors in 232 TAM treated breast cancer patients.

Factors	Integrin β_4_		
	Neg and %	Low and High Pos and %	P^0^
**Tumor size**			
T1	45 (25.6)	131 (74.4)	0.48
T2-T4	17 (30.4)	39 (69.6)	
**Lymph node status**			
Negative	48 (24.9)	145 (75.1)	0.16
Positive	14 (35.9)	25 (64.1)	
**Grading**			
G1	11 (20.8)	42 (79.2)	0.51
G2	41 (29.1)	100 (70.9)	
G3	10 (26.3)	28 (73.7)	
**ErbB-3**			
Negative	51 (33)	104 (67)	**0.003**
Positive	11 (14)	66 (86)	
**P-Akt (ser473)**			0.19
Negative	30 (31.6)	65 (68.4)	
Low	21 (22.8)	56 (77.2)	
High	11 (18)	49 (82)	
**ER β1**			
Negative	32 (43.2)	42 (56.8)	**<0.0001**
Positive	30 (19)	128 (81)	
**ErbB-2**			
Negative	50 (28.9)	123 (71.1)	0.40
Positive	12 (20.3)	47 (79.7)	

0 χ Test

### Impact of biopathological parameters on disease free survival

At a median follow up of 58 months (range 1–179 months), a total of 36 patients (15%) showed progressive disease.

The results of the univariate and multivariate analyses for DFS in the 232 patients included in this study are summarized in Table 3. Univariate analysis (Cox model) identified tumor size (HR 2.42, C.I. 1.25–4.68, p = 0.009), grading (G3, HR 4.78, C.I. 1.05–21.69, p = 0.04), nodal status (HR 2.25, C.I. 1.15–4.39, p = 0.018), ErbB-3 (HR 3.01, C.I. 1.56–5.82, p = 0.001), P-Akt overexpression (HR 5.03, C.I. 1.90–13.32, p = 0.001) and lack of ER β1 (HR 3.88, C.I. 1.98–7.59, p<0.0001) as significant predictors of DFS.

**Table 3:** Univariate and Multivariate analisyes of prognostic factors for Disease-Free Survival in 232 TAM treated breast cancer patients

	Univariate analysis		Multivariate analysis	
Factors	HR (95% CI)	p value	HR (95% CI)	p value
**Tumor size**				
>2 cm *vs*<2 cm	2.42 (1.25–4.68)	**0.009**	2.24 (1.10–4.53)	**0.02**
**Grading**				
G2 vs G1	3.19 (0.75–13.59)	0.12	5.61 (1.25–25.30)	**0.02**
G3 vs G1	4.78 (1.05–21.69)	**0.04**	10.23 (2.07–50.45)	**0.004**
G2 *vs* G3	0.67 (0.33–1.37)	0.27	0.55 (0.26–1.17)	0.12
**Nodal status**				
N_+_ vs N_0_	2.25 (1.15–4.39)	**0.018**		n.s.
**Integrin β_4_**				
positive *vs* negative	1.37 (0.63–2.95)	0.42		n.s.
**ErbB-3**				
positive *vs* negative	3.01 (1.56–5.82)	**0.001**	2.16 (1.10–4.22)	**0.024**
**P-Akt (ser473)**				
positive *vs* negative	5.03 (1.90–13.32)	**0.001**		n.s
**ERβ1**				
negative *vs* positive	3.88 (1.98–7.59)	**<0.0001**	3.28 (1.56–6.87)	**0.002**
**ErbB-2**				
positive *vs* negative	1.12 (0.55–2.28)	0.75		n.s.

Each variable that significantly affected DFS in the univariate analyses were introduced into a Cox proportional risk model. Multivariate analyses revealed that tumor size (HR 2.24, C.I. 1.10–4.53, p = 0.02), grading (G2 vs G1, HR 5.61, C.I. 1.25–25.30, p = 0.02 and G3 vs G1, HR 10.23, C.I. 2.07–50.45, p = 0.004), ErbB-3 expression (HR 2.16, C.I. 1.10–4.22, p = 0.024) and lack of ERβ1 (HR 3.28, C.I. 1.56–6.87, p = 0.002) were independent prognostic variables influencing DFS. ERβ1 negativity appears to be the most powerful prognostic indicator of a reduced DFS, indicating that ERβ1 positive tumors are more likely to be responsive to TAM therapy.

**Figure 5:**
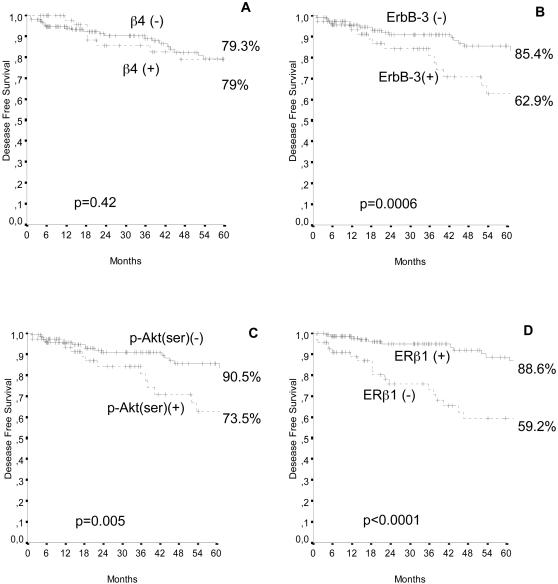
DFS (232 cases) for TAM treated patients with BC categorised on the basis of (A) β4, (B) ErbB-3, (C) P-Akt (ser473), (D) ERβ1 expression. Survival curves were generated according to the Kaplan-Meier method; statistical comparisons were made using the log-rank test.

Kaplan-Meier curves (Figure 5), stratified, respectively, for β4, ErbB-3, P-Akt and ERβ1 expression in all valuable cases, indicate that a significantly longer DFS can be observed in patients with ErbB-3 negative (p = 0.0006), P-Akt negative (p = 0.005) and ERβ1 positive (p<0.0001) tumors. β4 expression, considered as a single factor, did not influence the patient outcome.

**Figure 6:**
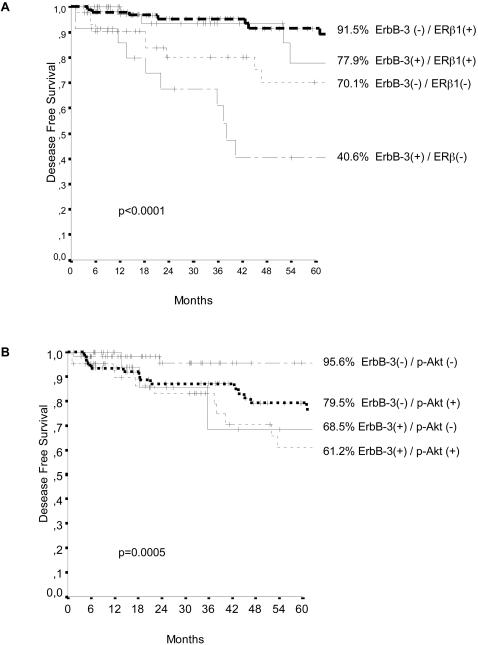
DFS (232 cases) for TAM treated patients with breast carcinomas categorised according to the combinations in all evaluated cases of (A) ErbB-3 and ERβ1 expression, (B) ErbB-3 and P-Akt expression. Survival curves were generated according to the Kaplan-Meier method; statistical comparisons were made using the log-rank test.

On the basis of these results, we evaluated the impact on DFS of β4, ErbB-3, P-Akt and ERβ1 combination. β4 expression, even if associated to the other three variables, did not add further useful clinical information. In contrast, as shown in Figure 6, the results obtained provide statistically significant evidence which indicates that the association of ErbB-3 positivity with ERβ1 negativity (p<0.0001) as well as the concomitant overexpression of p-Akt and ErbB3 (p = 0.0005) can identify subsets of patients with a high probability of relapsing within five years due to a worse response to TAM therapy.

## Discussion

It is widely known that α6β4 integrin expression and signaling are involved in the mechanisms that regulate tumor progression and resistance to apoptotic stimuli [4]–[9], [13], [15]. One of these mechanisms involves the ability of α6β4 integrin to regulate the translation of ErbB-3 receptor in a manner which is eIF-4E-dependent [15]. The ErbB-3 up-regulation associated with α6β4 integrin over-expression results in an increase of ErbB-2/ErbB-3 heterodimerization and consequent Akt phosphorylation favoring the survival of BC cells [15]. In the present work, we extended our previous study [15] to a novel panel of human BC cell lines which express different levels of α6β4 integrin confirming that the integrin expression correlates with ErbB-3 protein positivity. We also showed that β4 integrin depletion inhibits ErbB-3 translation and strongly reduces Akt activity while, ErbB-3 depletion abrogates Akt phosphorylation. Furthermore, the involvement of ErbB-3 in tumor progression was also supported by the finding that its depletion, in the absence of hormone stimuli, induces apoptosis, inhibits the *in vitro* invasion and favors TAM responsiveness. Given that ErbB-3 protein binds the regulatory subunit of PI3K but lacks kinase activity [24], our observations imply that ErbB-2/ErbB-3 sustains the survival of BC cells in the absence of ERs signaling through the activation of PI3K pathway. This hypothesis is strongly supported by recent findings which demonstrate that ErbB3 down-regulation by RNA interference abrogates ErbB-2-mediated TAM resistance in BC cells [25].

Our results are of particular clinical interest, since the anti-estrogen TAM plays a central role in the treatment of human BC. Nevertheless, many tumors appear to be refractory to TAM, making it necessary to discover predictive markers that can accurately identify hormone responsive tumors. In this setting, till the discovery of ERβ, ERα was the single most informative marker, receptor-negative tumors rarely benefiting from endocrine therapy [26]–[27]. In particular, it is important to note that cell-based studies have suggested that coexpression of ERβ in ERα-positive cells may modulate the ability of the cells to respond to estrogens [28]–[29] and studies using mice with targeted disruption of the ERβ gene have further supported this idea [30]. There is considerable evidence suggesting that for each action to block estrogen stimulation of BC cells, there are different reactions that tumor cells can adopt to escape the attempts to block their growth [31]. The activation of growth factor signaling is involved in the mechanism of resistance to endocrine therapy and it has been hypothesized that it may substitute estrogen in sustaining the growth and survival of BC cells [31].

Aimed at translating our in vitro results to human BC, we evaluated, by IHC, α6β4, ERβ1, ErbB3, and P-AKT expression in 232 primary mammary tumors derived from patients submitted to adjuvant TAM monotherapy. Even though we found a significant correlation between β4 and ErbB-3 expression and ERβ1 negativity, in the BCs we analyzed, the expression of the integrin did not influence the patient outcome.

ErbB-3 proteins mainly occurred in the P-Akt-positive and ERβ1-negative BC derived from patients with lower DFS. Although previous experimental studies have implied that α6β4 integrin facilitates tumor progression by regulating growth factor receptors signaling [15], to our knowledge this is the first study demonstrating an i*n vivo* correlation between β4 and ErbB-3 expression suggesting that β4 can regulate ErbB-3 protein *in vivo* and favor indirectly tumor progression.

The high percentage of mammary tumors we analyzed which over-express β4 integrin subunit is consistent with previous findings [8]. Although *in vivo* α6β4 integrin expression has not been extensively evaluated, there are two separate studies reporting that 90% of advanced BC expressed α6 subunit [32] and that high level of α6β4 expression in mammary tumor has prognostic value [33]. Furthermore, the over-expression of β4 integrin subunit in the ERβ1-negative tumors we have analyzed is also in agreement with a recent study which demonstrates that laminin-binding integrins and especially β4 integrin subunit is elevated in ER-negative BC [34].

These and numerous other studies conducted *in vivo* in a smaller number of tumors clearly indicate that β4 molecule mediates the signaling events which play a role in tumor progression [8]. This hypothesis is based on the capability of this integrin to enhance not only the translation of growth factor receptor but also of key growth factor such as VEGF [13], [35]. It has been observed that ablation of α6β4 expression by shRNA in BC cells impaired the ability of these cells to form xenograft tumors and to produce VEGF [13]. Moreover, the finding that the depletion of α6β4 integrin in mammary cells inhibits the PI3K pathway and facilitates the responsiveness to TAM treatment [12] correlates with the capability of α6β4 integrin to regulate ErbB-3 translation and subsequent Akt activation [15]. We can hypothesize that α6β4 integrin controls the translation of key molecules whose functions are strictly related to carcinoma survival. The ability of α6β4 integrin to control ErbB-3 expression *in vitro* [15] and the strong relationship between β4 and ErbB-3 receptor (P = 0.003, see Table 2) we observed *in vivo* confirms this hypothesis. Although in the BC we analyzed, β4 does not directly influence the patient outcome, its expression may influence a different regulation of ErbB-3 and consequently, as suggested by our analyses, PI3K activation through its heterodimerization with ErbB-2. Collectively, these phenotypic alterations may have a significant impact on DFS.

From our data it is evident that ErbB-3 may represent a key molecule involved in the mechanisms of TAM resistance in ERβ1-negative BC. This finding is in agreement with a recent report demonstrating that ErbB-3 modulates ErbB-2-mediated proliferation, colony formation and resistance to TAM treatment [25]. Even though there are many studies on the role of ErbB-2 in BC prognosis and therapeutic response, little is known regarding the role of ErbB-3 protein in these processes [18]. However, in agreement with our data, it has been found that the DFS is shorter in patients with ErbB-3 overexpression and that the level of ErbB-3 expression in primary BC seems to be involved in tumor progression from non-invasive to invasive tumors [36]. Moreover, it has also been shown that ErbB1-3 positive tumors had significantly poorer survival [37]. The strong relationship we found between ErbB-3 and P-Akt positivity and low DFS relative to patients with ERβ1-negativity reinforces the hypothesis that growth factor signaling is involved in the mechanism of resistance to endocrine therapy. However, from our study, it is clear that ERβ1 negativity appears to be the most powerful prognostic factor influencing DFS in response to TAM treatment and this data is in agreement with previous observations showing that low level of ERβ predict resistance to TAM treatment [38]. Together these studies provide strong evidence that ERβ1 is a predictor of response to TAM treatment in BC.

We can conclude that, even though the regulation of mammary tumor growth and survival by ERs and EGFR family members and the biology of β4 integrin in tumors are not completely known, our *in vitro* and *in vivo* results provide strong evidence of a functional cooperation among these factors in supporting the survival of mammary tumors and this cooperation in ERβ1-negative tumors may result in a decreased responsiveness to TAM therapy.

## Materials and Methods

### Cell lines

The human mammary carcinoma cell lines, MDA-MB361, BT474, SKBr3, MDA-MB231, T47D and BT549 were obtained from the ATCC and maintained in DMEM medium supplemented with 10% FCS (INVITROGEN, Milan, Italy). Rat bladder epithelial cell line 804G was cultured in minimum essential medium supplemented with 10% FCS and employed for LM5 rich matrix preparation [39].

### Antibody and matrix proteins

The rat anti-hum β4 subunit (Clone 439-9B) was prepared as previously described and used in immunoprecipitation, and immunofluorescence (FACS) analysis experiments [6]. The mouse anti-human β4 subunit 450-11A was used in western blotting and immunohistochemistry experiments [12]. The rabbit anti-ErbB-3 [Ab (C17), Ab6 (2B5)] and the mouse anti-ErbB-3 Ab (Ab-4 Clone H3.90.6) were used in western blot, immunoprecipitation and in immunofluorescence (FACS) analysis experiments, respectively. Clones C17 and 2B5 were purchased from Santa Cruz Biotechnologioes (Milan, IT) and clone Ab4 was purchased from NeoMarkers (Fremont, CA). The rabbit anti total and phospho-AKT (Ser473) antibodies were purchased from Cell Signaling (Milan, IT). The rabbit anti ERα and the mouse anti ERβ Abs were purchased from Santa Cruz Biotechnologioes (Milan, IT) and UCS Diagnostic (Rome, IT) respectively. The hsp70 (N27F3-4) Ab was purchased from Stressgen (Milan, IT). The mouse anti-PARP (Clone C2-10) was purchased from Pharmingen (Milan, Italy). FITC and Peroxidase-conjugated anti-IgGs were purchased from Cappel and BioRad (Milan, IT).The laminin-5-rich matrix from 804G cells was prepared as described previously [39]. In brief, 804G cells were plated onto 100 mm dishes or 96 well plates and allowed to reach confluence. The cells were washed in sterile PBS and were removed from their matrix by treatment for 10 min in 20 mM NH_4_OH at 4°C. The remaining cells were removed by washing three times with sterile PBS. The Poly-L-lysine was from SIGMA (Milan, Italy).

### Flow cytometry analysis

The expression level of β4, ErbB2 and ErbB-3 in MDA-MB 231, MDA-MB 361, BT474, SKBr3, BT549 and T47D cells was detected by flow cytometry analysis of stained cells. In brief, cells harvested using citrate saline buffer (0.134 M KCl, 0.015 M Na citrate) were washed twice with cold PBS containing 0.002% EDTA and 10 mM NaN_3_ (washing buffer). Samples of 1×10^6^ cells were incubated for 1 h at 4°C with saturating concentrations of primary antibodies diluted in PBS containing 0.5% bovine serum albumin (BSA). Cells were then washed three times with washing buffer (PBS containing 0.5% BSA) and incubated for 1 h at 4°C with 50 l of FITC-conjugated secondary antibodies [F(ab')2 (Cappel, West Chester, PA, U.S.A.)] diluted 1:20 in PBS/BSA. After three washes, the cells were suspended in 1 ml of washing buffer. Cell suspensions were analyzed by a flow-cytometer (Epics XL analyzer, Coulter Corporation, Miami, FL) after addition of 5l of a 1mg/ml solution of propidium iodide to exclude non-viable cells. At least 1×10^4^ cells per sample were analyzed.

### Western Blot analysis

To analyze ER-α, β4 and ErbB-3 protein expression, the cells were lysed with RIPA buffer (50 mM Tris (pH 8), 150 mM NaCl, 1% Nonidet P40, 0,1% deoxycholate, 0,1% SDS, 1mM PMSF, 5 mM Na_3_VO_4_, 50 mM protease inhibitors (SIGMA-Aldrich, Milan, IT) for 30 minutes at 4°C. Total cell lysates were clarified by centrifugation at 14,000 rpm for 30 minutes. Aliquots of cell extracts containing an equivalent amount of proteins were resolved by SDS-polyacrilamide gel electrophoresis 10% (SDS-PAGE) and transferred to nitrocellulose. To analyze Akt activation after stimulation by LM5, MDA-MB 361, BT474 and SKBr3 (1×10^6^) cell lines, after serum starvation for 24 hours, were seeded onto 100 mm tissue culture dishes coated with LM5-rich matrix preparation from 804G cells. The cells were washed three times with ice cold PBS and lysed with NP40 buffer (1% Nonidet P40, 10% glycerol, 137 mM NaCl, 20 mM Tris HCl (pH 7,4), 50 mM NaF, 1 mM PMSF, 5mM Na_3_VO_4, _50 mM protease inhibitors (SIGMA-Aldrich, Milan, IT) for 30 minutes at 4°C. Total cell lysates were clarified by centrifugation at 14,000 rpm for 30 minutes. Aliquots of cell extracts containing equivalent amounts of proteins were resolved by SDS-PAGE, transferred to nitrocellulose and probed with the rabbit polyclonal Ab directs to P-Akt. As secondary Abs, the horseradish peroxidase-coniugated goat anti-mouse or rabbit were used. The chemiluminescence was resolved by an enhanced chemiluminescence ECL kit (Amersham, Milan, IT). Total proteins were normalized by anti-actin, anti-Hsp70 and total-Akt Abs, respectively.

### RT-PCR

Total RNA was prepared using RNAzol B according to the manufacturer's procedure (Invitrogen, Milan, IT). Human ERβ1 mRNA for RT-PCR analysis was carried out using specific primers as previously described [40]. The oligonucleotides we use to amplify ERβ1 mRNA were as follow:

1. hERb1 sense: 5′TGCTTTGGTTTGGGTGATTGC3′;
2. hERβ1 anti-sense: 5′TTTGCTTTTACTGTCCTCTGC3′.

The housekeeping aldolase mRNA was used as an internal control.

### Immunocytochemistry

For the detection of ERβ by immunocytochemistry, 5×10^5^ cells of each cell line (MDA-MB 231, MDA-MB 361, SKBr3, BT474, BT549 and T47D) were centrifuged onto glass slides (cytospin) and fixed in 2% formaldehyde for 10 minutes. Endogenous peroxidase was blocked by incubating in 3% H_2_O_2_ in PBS for 10 minutes. After two rinses in PBS, nonspecific binding was blocked by a 10-minute incubation with normal serum (ScyTek Laboratories, Logan, UT). Samples were then incubated in mouse anti-ERβ1 antibody (1∶20 dilution) in 0,5% bovine serum albumine with PBS overnight, in a humidified atmosphere. Detection steps were done using the UltraTek HRP kit according to the manufacturer's procedure (ScyTek Laboratories), and peroxidase activity was localized with DAB (diamino-benzidine) substrate. Slides were counterstained by Hematoxilin and mounted under a coverslip in glycerol.

### RNA Interference

The inactivation of β4 was obtained by the LipofectAMINE PLUS™ method (INVITROGEN) using pSUPER.retro vector containing β4-shRNA or scramble RNA (scr-shRNA) sequences. To inactivate ErbB-3 expression, cells were transiently transfected with Transit-TKO reagent (MIRUS, Medison, Wisconsin) following the manufacturer procedures with the ErbB-3 anti-sense double strand siRNA as previously described [15]. The cells were harvested 48 hours post transfection with RIPA buffer for the detection of β4 and ErbB3 expression and with NP-40 buffer for the detection of P-AKT. Total proteins were separated by 8% and 10% SDS-PAGE respectively and transferred to nitrocellulose. The proteins were detected by western blot analysis as described above.

### Cell death and apoptosis

SKBr3, MDA-MB 361, BT474, TD47D and MDA-MB 231 cells (3×10^5^) were plated onto 60mm dishes in hormone-deprivation conditions for three days. The following day, the cells were trasiently transfected with a scrambled or ErbB-3 siRNA sequence and 24 hours after transfection the cells were treated with 2.5 µM TAM or ethanol as a control for 24 hrs. The viability of the cells was evaluated by Trypan blue exclusion. Each assay was repeated at least three times. Following the same procedure, the cells were lysed in Triton buffer (20 mM Tris pH 7.5, 150 mM NaCl, 1 mM EDTA, 1 mM EGTA, 1% Triton x-100, 0.5% NP40, 2.5% sodium pyrophosphatate, 1 mM Na_3_VO_4_, 50 mM protease inhibitors) and sonicated for 15 seconds. Samples were boiled for 5 minutes at 95°C, resolved by SDS-polyacrilamide gel electrophoresis (8%), transferred to nitrocellulose and probed with a mouse anti-PARP Ab.

### Chemoinvasion assay

Chemoinvasion was assessed using a 48-well modified Boyden's chamber (NeuroProbe, Pleasanton, CA) and 8-µm pore polyvinyl pyrrolidone–free polycarbonate Nucleopore filters (Costar, New York, NY). The filters were coated with an even layer of 3 mg/mL Cultrex (Trevigen, Gaithersburg, MD). The lower compartment of the chamber was filled with 24 hours conditioned serum free medium produced from NIH3T3 fibroblasts. SKBr3, MDA MB361, BT474, TD47D and MDA-MB 231 cells, after 3 days of hormone deprivation, were plated (1.5×10^6^ cells) onto 100 mm dishes. The following day, the cells were transfected with scrambled or ErbB-3 siRNA. Where specified, 24 hrs after transfection scrambled and ErbB-3 interferred cells were pre-incubated for 24 hours at 37°C with TAM 2.5 µM. The cells were, then, harvested (2×10^6^ cells/ml) and placed in the upper compartment (45 µl/well) of the Boyden's chamber. After 8 hours of incubation at 37°C, the cells migrated on the lower surface of the filters were fixed and stained with DiffQuick (Merz-Dade, Dudingen, Switzerland). Then, the migrated cells in 12 high-power fields were counted. Each assay was carried out in quadruplicate and repeated at least three times. The ability of the cells to adhere to the filters was verified by staining the upper side of the filter for each cell line.

### Patients

We studied a cohort of 232 hormonal receptor positive breast cancer patients surgically treated at the Regina Elena Cancer Institute (Rome, Italy) between 1986 and 2002, who had received an up-front adjuvant hormonal monotherapy with TAM at the dose of 20 mg per day for a maximum of 5 years. Invasive breast cancers were classified according to the World Health Organization Classification of Tumors [41] and were graded according to Bloom and Richardson. The information recorded for each patient consisted of: age at surgery, menopausal status, tumor size, axillary node status, histotype, and histologic grade. Patients selected for the study presented complete follow-up data and uniform methodology for hormone receptor content determination.

The study was reviewed and approved by the ethical committee of Regina Elena National Cancer Institute, and written informed consent was obtained from all patients.

### Immunohistochemistry

β4 integrin, ERβ1, P-AKT(ser473), ErbB-2 and ErbB-3 expression were assessed by indirect immunoperoxidase staining. Immunohistochemical staining was carried out on 5-µm-thick paraffin-embedded tissues. Sections were harvested on SuperFrost Plus slides (Menzel-Glaser, Braunschweig, Germany).

The deparaffinized and rehydrated sections were pretreated by microwave in 1mM citrate buffer (pH6.0) at 430 W (two 10′ cycles followed by a 5′ one) for ERβ1 and at 760 W (three 5′ cycles) for p-AKT(ser473), ErbB-2, ErbB-3 and β4 antigen.

Sections were incubated overnight with the anti-ERβ1 (clone PPG5/10, Biogenex,Space, Milan, Italy), the anti-β4 integrin (clone 450-11A directed to the cytoplasmic tail of the subunit) [12], the anti p-AKT(ser473) (Cell Signaling Technology, Sial, Rome, IT) and the anti-c-ErbB-3 (ErbB-3, clone RTJ-1, Novocastra Menarini, Florence, IT). ERβ1 was considered positive when more than 20% of neoplastic cells showed a nuclear immunoreactivity. ErbB-2 and ErbB-3 overexpression was determined as defined in the HercepTest kit guide (0 or 1+ negative, 2+ and 3+ positive).

The integrin β4 subunit was evidenced both in the membrane and in the cytoplasm of neoplastic cells and was scored considering both intensity and frequency from 0 to 2 according to the following criteria: 0. No Reaction, 1. Low Reaction (1–10% of positive cells with score +/++/+++ or >10–50% with score +), 2. High Reaction (>10–50% of positive cells with score ++/+++ or >50% with score +/++/+++). The P-AKT(ser473) immunostaining was scored as described for β4 protein.

The immunoreactions were revealed by a streptavidin-biotin-peroxidase system (Super Sensitive Link-Label IHC Detection System, Biogenex) using 3-amino-9-ethylcarbazole (Dako, Milan, IT) as a chromogenic substrate. All sections were slightly counterstained with Mayer's hematoxylin and mounted in aqueous mounting medium (UCS Diagnostics, Rome, IT). Evaluation of the immunohistochemical results was done independently and in blinded manner by two investigators (M.M, and P.A.).

### Statistical analysis

The correlation between β4 integrin expression and the biopathological characteristic variables was tested by the Pearson Chi-Square test. For the purpose of our study, disease-free survival (DFS) was considered as a measure of poor outcome. The disease free survival was calculated from the date of tumor diagnosis to the date of first recurrence or metastasis. Patients without recurrence were censored at the time of last follow-up or death. The Hazard risk and the confidence limits were estimated for each variable using the Cox univariate model and adopting the most suitable prognostic category as the referent group. The DFS curves were estimated by the Kaplan-Meier product-limit method. The log-rank test was used to assess differences between subgroups, and significance was defined as p<0.05.

A multivariate Cox proportional hazard model was also developed using stepwise regression (forward selection) with predictive variables which were significant in the univariate analyses. The enter limit and remove limit were p = 0.10 and p = 0.15, respectively. The SPSS (11.0) statistical program was used for analysis.

## Supporting Information

Figure S1 The expression of ERbeta1 protein was evaluated by immunocytochemistry. 5×105 MDA-MB 231, MDA-MB 361, SKBr3, BT474, BT549 and T47D cells were centrifuged onto glass slides and fixed in 2% formaldehyde for the dectection of ERb1 expression.
(10.39 MB TIF)

Figure S2 Representative invading stained cells. Chemoinvasion was assessed using a 48-well modified Boyden's chamber and 8-μm pore polyvinyl pyrrolidone-free polycarbonate filters. SKBr3, MDA MB361, BT474, TD47D and MDA-MB 231 (src, scr/TAM, B3si, B3si/TAM) cells migrated on the lower surface of the filters were fixed and stained. Then, the migrated cells in 12 high-power fields were counted. Each assay was carried out in quadruplicate and repeated at least three times.
(10.34 MB TIF)
