# Expression of Concern: Induction of ErbB-3 Expression by α6β4 Integrin Contributes to Tamoxifen Resistance in ERβ1-Negative Breast Carcinomas

**DOI:** 10.1371/journal.pone.0266300

**Published:** 2022-03-25

**Authors:** The PLOS ONE Editors

After this article [1] was published, concerns were raised about similarities between some lanes in western blot panels in Figs 1A, 1B, 2A and 3B.

Specifically:

- The α-actin blot data shown in lanes 5 and 6 of Fig 1A appear similar to the Akt data shown for BT474 cells in Fig 2A.
- In Fig 1B, there appear to be more similarities than would be expected between the bands shown in lanes 5 and 6 of the aldolase blot given the different samples.
- In Fig 3B, there appears to be a vertical discontinuity between lanes 2 and 3 of the BT474 PARP blot.

In response to the journal’s follow up on the above figures, the corresponding author indicated that they agree the α-actin blot data shown in lanes 5 and 6 of Fig 1A appears similar to the Akt data shown for BT474 cells in Fig 2A, and that a mistake may have been made during acquisition. The corresponding author stated that the bands in lanes 5 and 6 of the aldolase blot in Fig 1B appear similar as the experiment was performed using RT-PCR. They also stated that it is possible there is a vertical discontinuity between lanes 2 and 3 of the BT474 PARP blot in Fig 3B.

The underlying data supporting all figures and results in this article are no longer available.

Due to the unresolved concerns discussed above, the PLOS ONE Editors issue this Expression of Concern.
